# Subjective well-being among clinically stable psychiatric outpatients: differences between mood disorders, subthreshold conditions, and community controls

**DOI:** 10.1186/s12888-026-07927-z

**Published:** 2026-02-27

**Authors:** Mihoko Kawai, Hiroko Goji, Takahide Fukatsu, Jun Miyata, Kousuke Kanemoto

**Affiliations:** Department of Psychiatry, Aichi Medical University, 1-1 Karimata, Yazako, Nagakute, Aichi Japan

**Keywords:** Subjective well-being, Life satisfaction, Mood disorders, Subthreshold conditions, Psychiatric outpatients, Recovery

## Abstract

**Background:**

In modern psychiatric care, subjective well-being (SWB) has become an important indicator of recovery beyond symptom remission. However, it remains uncertain how SWB differs among clinically stable psychiatric outpatients and whether symptom stability necessarily reflects subjective recovery.

**Methods:**

This cross-sectional study compared SWB among clinically stable outpatients with mood disorders (M group; *n* = 59), a subthreshold group (ST group; *n* = 60), and community controls (CC; *n* = 204). The M group consisted of outpatients meeting DSM-5 criteria for depressive or bipolar disorders. The ST group comprised outpatients with persistent psychological distress—such as depressive mood, anxiety, and/or somatic complaints—who did not meet DSM-5 diagnostic criteria for any mood or anxiety disorders from initial assessment through enrollment. The CC group were recruited via an online survey panel. SWB was assessed using the Satisfaction with Life Scale (SWLS). Group differences were examined using covariance analysis, while predictors of SWB were explored using regression analysis.

**Results:**

After adjustment, a significant group effect on SWLS scores was observed. The M group showed significantly higher SWB than both the ST group and CC, whereas the ST group exhibited the lowest SWB, even though all outpatient groups met predefined criteria for clinical stability. Subjective depressive symptoms were strongly associated with lower SWB, while engagement in purpose-related activities was associated with higher SWB after adjustment.

**Conclusions:**

SWB among clinically stable psychiatric outpatients is heterogeneous and cannot be sufficiently explained by symptom severity or diagnostic categories alone. The relatively high levels of SWB observed in the M group may reflect the inclusion of long-term clinically stable outpatients, age-related differences, and adaptive psychological processes following illness, such as effective coping strategies and the recalibration of internal standards. In contrast, the ST group appeared to represent a clinically important help-seeking population in which subjective distress and psychological vulnerability persisted despite the absence of full diagnostic criteria. These findings underscore the clinical relevance of assessing SWB in outpatient psychiatric care and underscore the need for interventions that strengthen psychosocial resources related to meaning and purpose.

**Clinical trial number:**

Not applicable

**Supplementary Information:**

The online version contains supplementary material available at 10.1186/s12888-026-07927-z.

## Background

Individuals with mental disorders often require long-term treatment, with traditional psychiatric care primarily focusing on achieving stable symptom remission [1]. However, the World Health Organization defines mental health not merely as the absence of a mental disorder, but as a state of well-being that enables individuals to cope with stress, realize their abilities, work productively, and contribute to society [2]. From this perspective, psychiatric care has gradually shifted toward not only alleviating symptoms and suffering but also promoting meaningful and fulfilling lives for patients [1]. International discussions have further emphasized that outpatient and community mental health services should be evaluated not only for symptom control, but also for recovery-oriented outcomes such as well-being and social participation [3]. Recent studies in healthy populations have further demonstrated that higher subjective well-being (SWB) is associated with favorable life outcomes, underscoring its relevance in both public health and clinical contexts [4].

SWB refers to the extent to which individuals evaluate their own lives positively, encompassing both cognitive components, such as life satisfaction, and affective components, such as happiness [4]. Unlike quality of life (QOL), which emphasizes objective living conditions, SWB primarily reflects internal subjective experiences. Whereas QOL has been extensively examined in psychiatric populations, direct investigations of SWB remain relatively limited.

Importantly, SWB is conceptually distinct from both symptom severity and health-related QOL. Whereas health-related QOL primarily reflects perceived functional limitations and overall health status, SWB encompasses broader dimensions of lived experiences, including meaning, purpose, and overall life satisfaction. From a psychiatric rehabilitation perspective, these dimensions are clinically relevant, as recovery is increasingly understood not only in terms of symptom reduction but also as the capacity to lead a personally meaningful and fulfilling life. Thus, SWB represents a patient-centered outcome that complements traditional clinical and functional measures, particularly in the context of long-term outpatient care.

Previous studies have consistently reported that individuals with mental disorders tend to experience lower SWB than community control groups [5–11]. Moreover, little is known about how diagnostic thresholds influence patients’ subjective life evaluations in routine outpatient settings, particularly within health systems characterized by long-term follow-up. Although depressive symptoms have consistently been identified as important correlates of SWB, the contributions of socioeconomic and psychosocial factors have not been systematically examined across diagnostic groups. Consequently, despite the marked heterogeneity of psychiatric outpatients, few studies have conducted comprehensive cross-group comparisons, limiting the development of patient-centered care strategies that address well-being beyond symptom reduction.

Furthermore, recent research has indicated that subthreshold symptom groups—those experiencing clinically meaningful emotional distress despite not meeting full diagnostic criteria—should not be regarded as merely mild conditions, as they are often associated with substantial functional impairment and reduced QOL [12–14]. Epidemiological studies suggest that subthreshold depressive and anxiety symptoms are highly prevalent, affecting approximately 15–25% of the general population and an even higher proportion of help-seeking individuals in mental health services [12, 13]. These presentations have been linked to meaningful functional burden, including occupational impairment and increased health service utilization and are associated with an elevated risk of progression to full-threshold mood or anxiety disorders [13]. Moreover, subthreshold symptoms of depression, anxiety, and somatic complaints frequently co-occur and overlap [14]. Despite their clinical relevance in outpatient psychiatry, few studies have directly compared such heterogeneous subthreshold groups with patients meeting full diagnostic criteria or community controls. In the present study, the subthreshold (ST) group was conceptualized as a clinically relevant help-seeking population characterized by persistent subjective distress that did not meet categorical Diagnostic and Statistical Manual of Mental Disorders, Fifth Edition (DSM-5) diagnostic criteria.

The present study aimed to compare SWB among stably treated, community-dwelling outpatients with mood disorders (M group), the ST group, and community controls (CC), and to examine how SWB is independently associated with key sociodemographic (e.g., age, education, income, living conditions), clinical (e.g., illness duration, depressive symptom severity), and psychosocial variables reflecting social context and meaning-related engagement (e.g., social support and engagement in purpose-related activities) across these groups. Based on previous literature, we hypothesized that the M group would exhibit levels of SWB comparable to those of CC, whereas individuals in the ST group would demonstrate lower SWB. We further hypothesized that psychosocial factors reflecting social context and engagement in purpose-related activities would serve as significant independent predictors of SWB, beyond sociodemographic and clinical variables.

## Methods

### Study design and participants

This study employed a cross-sectional design. A total of 323 participants were recruited, including 59 outpatients with mood disorders (M group; depressive disorder, *n* = 25; bipolar disorder, *n* = 34), 60 outpatients in the subthreshold group (ST group), and 204 community controls (CC).

Outpatient participants were recruited from Aichi Medical University Hospital, Toyota Welfare Hospital, and Suzukake Clinic between June 2021 and March 2023. The inclusion criteria for the outpatients were as follows:

1. Age ≥ 20 years (observed range: 22–88 years).
2. Currently attending regular outpatient care and able to maintain independent living in the community.
3. Classified into the following diagnostic subgroups at the time of assessment, with diagnoses reviewed longitudinally during routine clinical follow-up and finalized at the time of enrollment:

- M group: outpatients who met the DSM-5 criteria for depressive or bipolar disorder, with a confirmed history of at least one depressive episode.
- ST group: outpatients who sought psychiatric care due to persistent subjective distress (e.g., depressed mood, anxiety, and/or somatic complaints) but did not meet DSM-5 diagnostic criteria for any current mood or anxiety disorder at initial psychiatric assessment or during follow-up before enrollment, despite receiving regular outpatient psychiatric treatment.

Diagnostic status and group classification were determined by board-certified psychiatrists with more than 5 years of clinical experience, using DSM-5 criteria and the Mini International Neuropsychiatric Interview [15]. Diagnostic decisions were routinely reviewed through clinical case discussions as part of standard psychiatric practice to ensure consistency and diagnostic accuracy.

All outpatients were required to have:

- no hospitalization due to symptom exacerbation within the past 6 months,
- no major changes in medication within the past 3 months, and.
- a Clinical Global Impression-Severity (CGI-S) score of < 4.

These criteria were designed to minimize the influence of acute symptom exacerbations, treatment-related fluctuations, and comorbid conditions that might independently affect SWB, thereby enabling a more focused examination of SWB in relatively stable outpatient settings. CC were recruited online via Rakuten Insight, matched with outpatients by age and sex. This study was approved by the Ethics Committee of Aichi Medical University (Approval No. 2021-031) and was conducted in accordance with the Declaration of Helsinki. Written informed consent was obtained from all participants prior to enrollment. All data were anonymized before analysis.

### Variables and data collection

Demographic and clinical data were collected through interviews and medical records, including age, sex, living situation, marital status, presence of children, education level, employment status, occupation, and household income. Household income was categorized into two groups (below and above 5 million yen), based on the approximate national average in Japan [16]. Purpose-related activities were assessed using a single self-report binary item asking whether participants engaged in hobbies or activities they perceived as meaningful (yes/no). Interactions with friends were assessed using a single binary item asking whether participants regularly met with or communicated with friends. Perceived social support was measured with a single-item measure reflecting the perceived availability of support. social networking service (SNS) use was assessed with a single binary item asking whether participants regularly used SNS platforms. Because all of these constructs were assessed using single-item measures, Cronbach’s alpha was not applicable. Information on age at onset, duration of illness, and medication history was obtained from medical records.

### Clinical measurements

SWB was assessed as part of a clinical evaluation using the Japanese version of the satisfaction with life scale (SWLS) [17]. The reliability and validity of the SWLS have been widely confirmed across various sociodemographic and clinical populations [18]. This self-reported measure consists of 5 items rated on a 7-point Likert scale (1 = strongly disagree to 7 = strongly agree), with higher scores indicating greater life satisfaction. The total score ranges from 5 to 35 [17]. The scale also exhibits a stable factor structure across diagnostic groups, supporting its applicability for comparing SWB among individuals with various mental disorders [7].

The Japanese version of the Brief Psychiatric Rating Scale was used to evaluate the psychopathological severity of the participants in the outpatient group [19, 20]. This 18-item psychiatric symptom rating scale assesses various mental symptoms without specific disease limitations. Several studies have confirmed its psychometric properties, including reliability, validity, and sensitivity [21].

The Japanese version of the Patient Health Questionnaire 9 (PHQ-9) was administered to evaluate subjective feelings of depression [22]. This self-report tool evaluates the presence and severity of nine core symptoms, including depressed mood, loss of interest, and insomnia. Its validity as a screening tool, including its psychometric reliability, has been widely confirmed in different countries [23].

In the present sample, internal consistency was good for the SWLS (Cronbach’s α = 0.870) and excellent for the PHQ-9 (α = 0.887).

The standard Japanese edition of the Short Form 12 Health Survey (SF-12) version 2 was used to assess health-related QOL, with a particular focus on physical health components [24]. The SF-12 is a shortened version of the SF-36 [25] and consists of 12 items designed to evaluate health-related QOL in both clinical and non-clinical populations [26]. In the present study, the physical functioning (PF), role physical (RP), bodily pain (BP), and general health perceptions (GH) subscales were used to characterize participants’ physical health status. Scores were calculated using norm-based scoring and interpreted with reference to Japanese national normative values for descriptive purposes.

### Statistical analysis

Statistical analyses were conducted using IBM SPSS Statistics for Windows, version 30.0 (IBM Corp., Armonk, NY, USA).

First, demographic and clinical characteristics were summarized and compared across the M group, ST group, and CC. Categorical variables were analyzed using chi-square tests, and continuous variables were compared using one-way analysis of variance (ANOVA).

Second, differences in SWB (SWLS scores) among the three groups were examined using analysis of covariance (ANCOVA), adjusting for a priori selected covariates based on clinical relevance and prior literature, including depressive symptom severity (PHQ-9), in order to determine whether group differences in SWB persisted beyond current depressive symptom levels. The assumption of homogeneity of the regression slopes was tested, and no significant group-by-covariate interactions were observed. Both unadjusted and adjusted group comparisons were conducted, and post hoc pairwise comparisons of estimated marginal means were performed using Bonferroni correction.

Third, multiple linear regression analyses were conducted within the outpatient sample to identify independent predictors of SWB. Regression models included clinically relevant and potentially modifiable variables, while demographic factors already controlled for in the ANCOVA were not re-entered. Covariates were selected a priori based on clinical relevance to avoid overadjustment. Because depressive symptom severity conceptually overlaps with SWB, PHQ-9 scores were excluded from the multivariable regression models. Variables such as employment status and physical health conditions were not included in the primary models, as physical health was characterized descriptively using SF-12 scores and employment status was examined separately in descriptive analyses. Given the outpatient sample size (*n* = 119) and conceptual overlap among single-item psychosocial indicators, we adopted a parsimonious model that included variables representing core social context (living with someone, perceived social support) and meaning-related engagement (purpose-related activities), thereby minimizing risks of overfitting and multicollinearity.

Assumptions of normality, homoscedasticity, and multicollinearity were assessed using residual plots, variance inflation factors, and tolerance statistics; no significant violations were detected. Missing data were minimal and handled using complete case analysis. For all analyses, a two-tailed p-value < 0.05 was considered statistically significant.

Finally, a sensitivity power analysis was conducted using G*Power 3.1 for one-way ANOVA (fixed effects, omnibus) with three groups (total *N* = 323). With a significance level of *α* = 0.05 and power (1 − *β*) = 0.80, the minimum detectable effect size was Cohen’s *f* = 0.185. This indicates that the present sample size was sufficient to detect small-to-moderate overall group differences in SWLS scores. Because ANCOVA included covariate adjustment, the effective power for detecting group differences is expected to be comparable to or greater than this conservative estimate.

## Results

### Outpatient and CC characteristics

Overall, 59 outpatients in the M group, 60 outpatients in the ST group, and 204 CC were included in the analysis. All outpatients were receiving regular psychiatric care and met predefined clinical stability criteria, including no recent hospitalization, no major medication changes, and low clinician-rated symptom severity (CGI-S < 4). Within the M group, 34 patients were diagnosed with bipolar disorder, and 25 with depressive disorder. The main sociodemographic and clinical characteristics are summarized in Table 1, with more detailed sociodemographic characteristics provided in a table in Additional file 1.

**Table 1:** Sociodemographic and clinical features and clinical ratings of the three groups

Variable	M group (*n* = 59)	ST group (*n* = 60)	CC (*n* = 204)	*p*
Age, years, *m* (SD)	62.3 (15.30)	46.8 (13.80)	48.9 (14.10)	< 0.001
Sex, male, *n* (%)	20 (33.9)	13 (21.7)	67 (32.8)	0.223
Physical illness, *n* (%)	32 (54.2)	25 (36.6)	66 (32.4)	< 0.05
Living with someone, *n* (%)	50 (84.7)	56 (93.3)	179 (87.7)	0.326
Married, *n* (%)	39 (66.1)	34 (56.7)	137 (67.2)	0.320
Has children, *n* (%)	49 (83.1)	30 (50.0)	85 (41.7)	< 0.001
Education, years, *m* (SD)	13.5 (2.33)	13.9 (1.79)	14.2 (2.01)	0.086
Unemployed, *n* (%)	17 (28.8)	16 (26.7)	23 (11.3)	< 0.01
Homemaker, *n* (%)	21 (35.6)	16 (26.7)	34 (16.7)	< 0.05
Part-time, *n* (%)	5 (8.5)	17 (28.3)	45(22.1)	< 0.05
Full-time, *n* (%)	16 (27.1)	11 (18.3)	102 (50.0)	< 0.001
Household income (< 5 million yen/year), *n* (%)	42 (71.2)	41 (68.3)	82 (40.2)	< 0.001
Interactions with friends, *n* (%)	50 (84.7)	52 (86.7)	193 (94.6)	< 0.05
Social support, *n* (%)	51 (86.4)	48 (80.0)	170 (83.3)	0.662
Purpose-related activities, *n* (%)	42 (71.2)	36 (60.0)	161 (78.9)	< 0.05
Uses SNS, *n* (%)	11 (18.6)	32 (53.3)	139 (68.1)	< 0.001
PHQ-9, *m* (SD)	4.8 (4.81)	11.5 (6.92)	6.0 (5.17)	< 0.001
SF-12 PF, *m* (SD)	44.8 (15.40)	42.6 (14.30)	-	-
SF-12 RP, *m* (SD)	45.2 (12.80)	40.1 (14.50)	-	-
SF-12 BP, *m* (SD)	47.0 (13.40)	39.2 (15.90)	-	-
SF-12 GH, *m* (SD)	50.0 (9.50)	40.0 (10.00)	-	-
BPRS ^a^, *m* (SD)	22.2 (4.20)	23.0 (3.10)	-	0.231
Illness duration ^a^, *m* (SD)	13.5 (12.50)	11.6 (8.70)	-	0.319
Antidepressant use ^a^, *n (%)*	23 (38.9)	14 (23.3)	-	0.065

Note. Values are presented as mean (SD) unless otherwise indicated. p-values indicate overall group effects derived from one-way ANOVA for continuous variables and *χ²* tests for categorical variables, where applicable. Post hoc comparisons are described in the text. SF-12 scores are shown for descriptive purposes only, without inferential statistical testing. M group = mood disorders group; ST group = subthreshold group; CC = community controls; SWLS = Satisfaction with Life Scale; SNS = social networking service; PHQ-9 = Patient Health Questionnaire–9; SF-12 = Short Form–12 Health Survey; PF = physical functioning; RP = role physical; BP = bodily pain; GH = general health perceptions; BPRS = Brief Psychiatric Rating Scale

^a^Patients only

Significant group differences were observed in age, physical illness, parenthood, employment status, household income, interactions with friends, engagement in purpose-related activities, and SNS use. Post hoc analyses indicated that both outpatient groups (M and ST) had higher rates of unemployment and lower levels of full-time employment and household income than CC. In addition, the M group exhibited a higher prevalence of physical illness and parenthood, as well as fewer interactions with friends. In contrast, the ST group was characterized by lower engagement in purpose-related activities.

No significant group differences were found in sex, marital status, living arrangement, or perceived social support. Regarding treatment status, most outpatients were receiving ongoing psychiatric care, including pharmacotherapy and/or structured psychotherapy, consistent with routine clinical practice. Antidepressant use was more frequent in the M group than in the ST group (38.9% vs. 23.3%), although this difference did not reach statistical significance (*χ²* = 3.40, *p* = .065).

### Comparison of SWLS between groups

In the unadjusted analyses, mean SWLS scores differed significantly among the three groups (one-way ANOVA, (*F*(2, 320) = 25.30, *p* < .001), with the M group exhibiting higher scores than both the ST group and CC.

After adjusting for age, living status, and PHQ-9, ANCOVA revealed a significant main effect of group on SWLS scores (*F*(2, 317) = 9.19, *p* < .001, partial *η²* = 0.055). Among the covariates, only PHQ-9 scores were significantly associated with SWLS (*p* < .001), whereas age and living status were not. The assumption of homogeneity of regression slopes was satisfied.

**Fig. 1:**
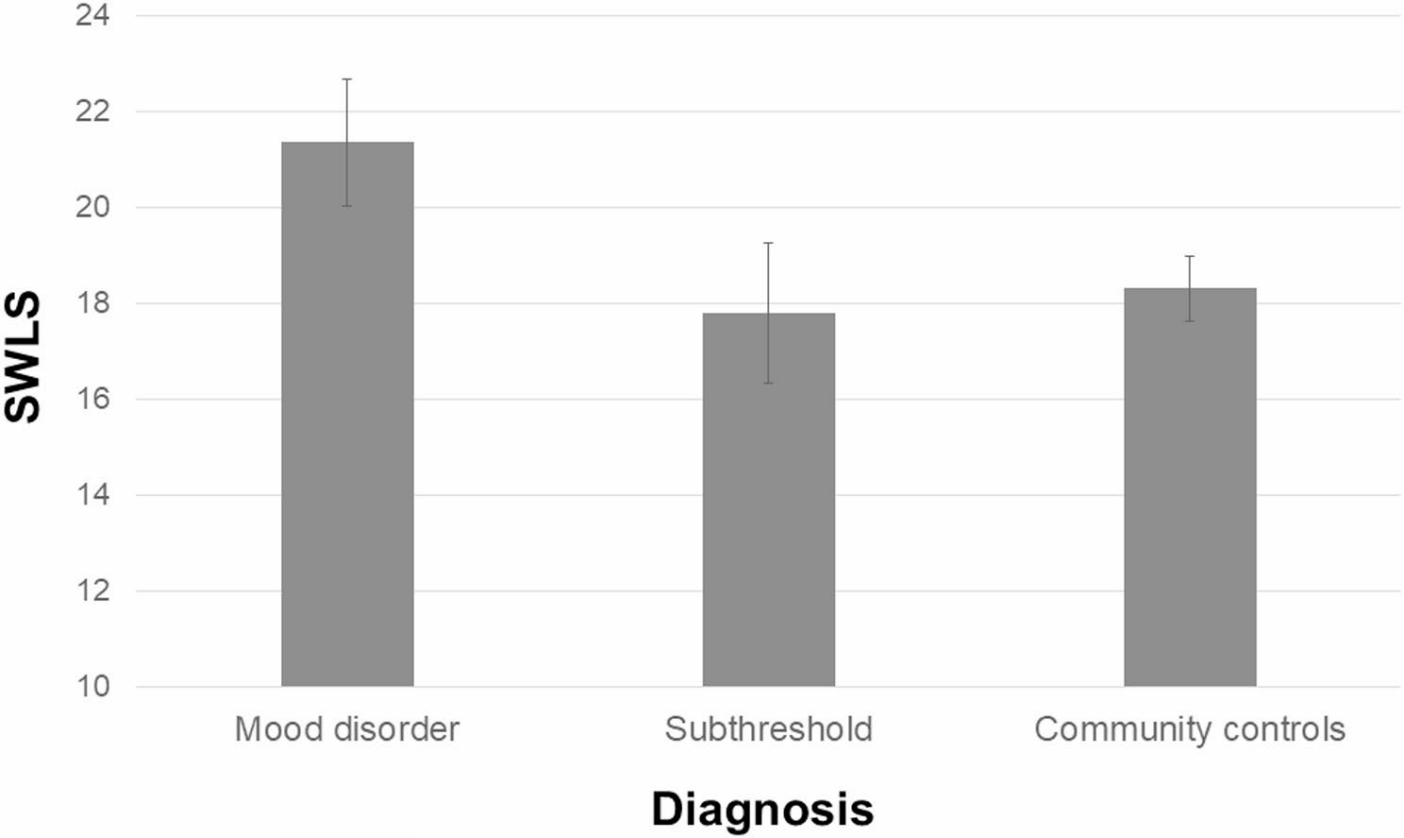
Comparison of Satisfaction with Life Scale (SWLS) scores across study groups. Values represent estimated marginal means with 95% confidence intervals, derived from ANCOVA adjusted for clinically relevant covariates

Bonferroni-corrected post hoc comparisons based on estimated marginal means indicated that the M group had significantly higher SWLS scores than both the CC (*p* < .001) and the ST group (*p* < .001). No significant difference was observed between the ST group and CC (*p* = 1.000). Unadjusted and adjusted SWLS means are presented in Table 2. Adjusted pairwise comparisons with Bonferroni correction are also shown, and adjusted SWLS scores across the three groups are illustrated in Fig. 1.

**Table 2:** Adjusted means of SWLS and ANCOVA results across groups

(A)Adjusted means of SWLS										
Group	SWLS mean (SD)			Adjusted mean				SE		95% CI
M group	22.53 (5.43)			21.36				0.66		[20.05, 22.66]
ST group	15.48 (5.51)			17.65				0.66		[16.34, 18.96]
CC	18.68 (5.37)			18.38				0.34		[17.71, 19.05]

(B)Overall ANCOVA results										
Effect			*F* (df)			*p*			η²p	
Group			*F*(2, 317) = 9.19			< 0.001			0.055	

(C)Post hoc pairwise comparisons (Bonferroni-adjusted)										
Comparison		Mean difference			SE		*p*			
M vs. CC		2.98			0.75		< 0.001			
M vs. ST		3.71			0.97		< 0.001			
ST vs. CC		0.73			0.76		1.000			

Note. Unadjusted group differences were examined using one-way ANOVA (*F*(2, 320) = 25.30, *p* < .001). Adjusted means were estimated using ANCOVA, controlling for age, living status, and depressive symptoms (PHQ-9). Bonferroni correction was applied for post hoc comparisons. SWLS = Satisfaction with Life Scale; SE = standard error; CI = confidence interval; M group = mood disorders group; ST group = subthreshold group; CC = community controls

### Other clinical measurements

#### PHQ-9

PHQ-9 scores differed significantly among the three groups (Welch’s *F*(2, 109.83) = 19.99, *p* < .001). The ST group showed the highest level of depressive symptoms (mean = 11.50, SD = 6.92), followed by community controls (mean = 6.04, SD = 5.17) and the M group (mean = 4.81, SD = 4.73). Post hoc comparisons using the Games–Howell procedure indicated that PHQ-9 scores were significantly higher in the ST group than in both the M group and community controls (both *p* < .001), whereas no significant difference was observed between the M group and community controls. Notably, PHQ-9 scores among community controls were higher than those typically reported in population-based samples, reflecting the absence of structured diagnostic screening in this group. Group differences in PHQ-9 are summarized in Table 1.

#### SF-12

Descriptively, both outpatient groups demonstrated lower physical health–related SF-12 scores than CC across PF, RP, BP, and GH domains. The ST group tended to show poorer BP and GH scores than the M group. No consistent differences were observed between the M and ST groups in PF or RP. Detailed group comparisons are shown in Table 1.

#### BPRS

When comparing the two patient groups, total BPRS scores did not significantly differ (mean ± SD: 22.15 ± 4.17 vs. 22.97 ± 3.15, *p* = .23), indicating comparable levels of clinician-rated psychopathology. These results are summarized in Table 1.

### Associations between clinical and psychosocial variables and SWLS

To examine factors independently associated with SWB, a multiple linear regression analysis was conducted in the outpatient sample (*n* = 119), with SWLS score as the dependent variable. The overall model was statistically significant (*R* = .544, adjusted *R²* = 0.271, *F*(4,114) = 11.97, *p* < .001). In the multivariable model, older age (*β* = 0.42, *p* < .001) and engagement in purpose-related activities (*β* = 0.24, *p* = .006) were independently associated with higher SWLS scores. Living with someone showed a trend-level association with SWLS (*β* = 0.14, *p* = .078), whereas perceived social support was not significantly associated with SWLS (*p* = .224). Results of the multiple regression analysis are presented in Table 3.

**Table 3:** Multiple regression analysis predicting SWLS scores in the outpatient group (*n* = 119)

Predictor	B	SE	β	t	*p*
Age	0.166	0.031	0.42	5.31	< 0.001
Living with someone	2.944	1.655	0.14	1.78	0.078
Social support	1.776	1.452	0.10	1.22	0.224
Purpose-related activities	3.177	1.128	0.23	2.82	0.006

Model fit: *R²* = 0.296, adjusted *R²* = 0.271, F(4, 114) = 11.97, *p* < .001

Note. B = unstandardized coefficient; SE = standard error; *β* = standardized coefficient; SWLS = Satisfaction with Life Scale. All predictors were entered simultaneously (forced-entry method)

## Discussion

This study identified distinct group differences in SWB among clinically stable psychiatric outpatients, indicating that SWB cannot be fully explained by depressive symptom severity or clinician-rated psychopathology alone. Contrary to our initial hypothesis of equivalence between the M group and CC, the M group demonstrated significantly higher SWB after covariate adjustment. Most notably, the M group exhibited significantly greater life satisfaction than both the ST group and CC, even after adjustment for self-reported depressive symptoms. This pattern underscores that SWB represents a distinct dimension of recovery that cannot be inferred linearly from diagnostic status or residual symptom severity alone. These findings contrast with previous reports suggesting persistently reduced well-being in individuals with mood disorders, including those in remission [8, 11]. Importantly, both outpatient groups were clinically stable and receiving routine psychiatric care without recent hospitalization or intensive interventions, and clinician-rated psychopathology severity was comparable between the M and ST groups. Taken together, these observations suggest that the observed differences in SWB are unlikely to be sufficiently explained solely by variations in treatment intensity or overall clinical severity.

Several factors may account for the relatively high levels of SWB observed in the M group. Previous studies reporting persistently reduced well-being in individuals with mood disorders have often focused on patients in the acute phase or early recovery period, or have not clearly specified the duration of symptom stability [8, 11]. In contrast, the present study targeted outpatients who had maintained clinical stability over a sustained period, as reflected by the absence of recent hospitalization, stable medication regimens, and low clinician-rated symptom severity. Consistent with longitudinal evidence indicating that mental health–related quality of life can improve following depressive episodes [27], these findings suggest that, among outpatients with sustained clinical stability, SWB does not necessarily remain diminished.

This pattern may also reflect psychological adaptation processes not fully captured by symptom stability alone. Experiencing periods of clinically significant mood symptoms followed by stabilization may serve as a psychological reference point, enabling individuals to reappraise their current life more positively. In the context of long-term outpatient care, patients with mood disorders may gradually recalibrate their expectations and personal definitions of recovery, a process often described as response shift [28]. Related perspectives in the literature suggest that recovery from significant illness or adversity can be accompanied by positive changes in life appraisal or meaning-making [29]. Taken together, these perspectives may help explain why the M group reported higher SWB despite lower employment rates, household income, and physical health compared with community controls, a pattern that contrasts with findings in the general population, where SWB is typically strongly associated with external resources [4, 30].

Despite these plausible explanations, this interpretation should be approached with caution. Although the influence of manic symptoms cannot be entirely excluded, clinician-rated assessments using the BPRS did not indicate a marked elevation in manic symptoms within the M group. Additionally, alternative explanations related to study design should be acknowledged, including selective retention of relatively higher-functioning individuals in long-term outpatient care, age-related effects, and potential response biases. Importantly, even after adjusting for age and depressive symptom severity, the elevated SWB observed in the M group remained significant, suggesting that factors beyond symptom burden alone may contribute to SWB in this population.

In contrast, the ST group exhibited the lowest levels of SWB, despite meeting predefined criteria for clinical stability and living independently in the community. This group reported the highest levels of self-rated depressive symptoms, whereas clinician-rated psychopathology did not significantly differ from that of the M group. This dissociation highlights a limitation of categorical diagnostic thresholds: clinically meaningful distress may not be adequately captured when individuals fall below formal diagnostic criteria. Editorial perspectives have similarly emphasized that substantial subjective burden may persist even in clinically stable or treatment-responsive cases, underscoring the need for outcome frameworks that extend beyond symptom-based definitions of recovery [31]. Consistent with prior population-based and longitudinal studies, subthreshold psychiatric symptoms have been shown to predict subsequent functional impairment, increased healthcare utilization, and an elevated risk of developing diagnosable mood disorders [12, 14]. Furthermore, meta-analytic evidence indicates that psychological interventions may be beneficial even in the absence of a full-threshold diagnosis [32]. Accordingly, subthreshold conditions should not be regarded as merely milder forms of mood disorders, but rather as a clinically distinct help-seeking population characterized by persistent subjective distress and reduced engagement in daily life.

Notably, unlike the ANCOVA models examining group differences, depressive symptom severity (PHQ-9) was not included as an independent variable in the regression analyses to avoid overadjustment, given its conceptual overlap with SWB. Multivariable analyses further indicated that SWB among psychiatric outpatients was independently associated with engagement in purpose-related activities, even after accounting for basic demographic and social context variables. This finding suggests that involvement in meaningful or purpose-oriented activities may represent an important psychological resource contributing to SWB beyond basic social circumstances alone. Although purpose-related activities were assessed using a brief behavioral indicator rather than a dedicated psychometric scale, similar associations between engagement in valued activities and SWB have consistently been reported in both general and clinical populations [3, 33]. The present findings extend this literature by underscoring the relevance of such purpose-related resources among clinically stable psychiatric outpatients.

From a clinical perspective, these findings suggest that symptom stability or the absence of a full-threshold diagnosis should not be equated with recovery, particularly among patients with subthreshold symptoms. This view is consistent with international discussions emphasizing that the organization of outpatient and community mental health services plays a critical role in promoting recovery and well-being beyond symptom control [3]. Reliance solely on clinician-rated symptom severity may lead to under-recognition of persistent subjective distress and low well-being. Greater attention to patients’ subjective experiences and engagement in purpose-related activities may therefore be warranted. This interpretation aligns with contemporary recovery-oriented frameworks, which emphasize meaning, agency, and participation in valued life activities beyond symptom remission [34]. Routine assessment of SWB using brief measures of life satisfaction in outpatient settings may facilitate the identification of individuals who remain vulnerable despite clinical stability and inform the provision of low-intensity psychosocial or meaning-oriented interventions.

Several limitations should be acknowledged. First, the cross-sectional design precludes causal inference, and SWB was assessed using self-report measures. The use of a clinically stable outpatient sample may limit the generalizability of the findings to more acutely ill or unstable psychiatric populations. Second, community controls were recruited via an online panel and were not screened using structured diagnostic interviews; therefore, some participants may have had clinically relevant depressive symptoms, limiting strict interpretation of comparisons with clinical groups. Third, although the ST group was operationally defined, diagnostic heterogeneity remains, and residual or prodromal conditions cannot be fully excluded. Additionally, individual vulnerability factors, including personality-related characteristics, may contribute to persistent distress in some ST patients; however, such factors were not systematically assessed in this study. Fourth, purpose-related activities were operationalized using a single-item behavioral indicator rather than a validated multidimensional scale, which may limit interpretive precision. Finally, SF-12 physical health scores were interpreted descriptively using norm-referenced scoring, without inferential statistical comparisons. Although the overall sample size provided sufficient statistical power to detect omnibus group differences, this study may have been underpowered to identify very small differences between the two outpatient subgroups. Despite these limitations, the present findings yield clinically meaningful insights into SWB among stably treated psychiatric outpatients and highlight the importance of recovery-oriented outcomes that extend beyond symptom remission in routine outpatient care.

## Conclusion

In conclusion, the present findings suggest that SWB represents a dimension of recovery that is not fully captured by depressive symptom severity or clinician-rated psychopathology. While individuals with mood disorders with sustained clinical stability may demonstrate relatively high levels of SWB, patients with subthreshold symptoms may remain psychologically vulnerable despite apparent clinical stability. Further longitudinal and interventional research is warranted to elucidate how engagement in purpose-related activities and SWB interact with symptom trajectories and to inform more comprehensive recovery-oriented approaches in routine outpatient psychiatry.

## Supplementary Information

Below is the link to the electronic supplementary material.

Supplementary Material 1: Supplementary statistical results and descriptive statistics.

## Data Availability

Data are available from the corresponding author upon reasonable request.

## Declarations

### Generative AI and AI-assisted technologies

During the preparation of this manuscript, the authors used ChatGPT (OpenAI) for language refinement. The authors reviewed and edited all generated content and take full responsibility for the final manuscript.

## Abbreviations

ANOVA: Analysis of variance
BP: Bodily pain
CC: Community controls
CGI-S: Clinical Global Impression-Severity
DSM-5: Diagnostic and Statistical Manual of Mental Disorders, Fifth Edition
GH: General health
M: Mood Disorders group
PF: Physical functioning
PHQ-9: Patient Health Questionnaire-9
QOL: Quality of life
RP: Role physical
SF-12: Short Form Health Survey 12
SNS: Social networking service
SPSS: Statistical Package for the Social Sciences
ST: Subthreshold group
SWB: Subjective well-being
SWLS: Satisfaction with life scale
