# Supplementary material for: Subjective well-being among clinically stable psychiatric outpatients: differences between mood disorders, subthreshold conditions, and community controls
Source: BMC Psychiatry. 2026 Feb 27;26:218. doi: 10.1186/s12888-026-07927-z (PMC12952017; doi:10.1186/s12888-026-07927-z)
Supplement: Supplementary file 1 — Supplementary Material 1: Supplementary statistical results and descriptive statistics. [file 12888_2026_7927_MOESM1_ESM.docx]

| Variable | M group  (n = 59) | ST group  (n = 60) | CC  (n = 204) | *Statistic (df)* | *p* |
| --- | --- | --- | --- | --- | --- |
| Sex, male, *n* (%) | 20 (33.9) | 13 (21.7) | 67 (32.8) | *χ²(*2) = 3.00 | *p* = .223 |
| Living with someone, *n* (%) | 50 (84.7) | 56 (93.3) | 179 (87.7) | *χ²*(2) = 2.24 | *p* = .326 |
| Married, *n* (%) | 39 (66.1) | 34 (56.7) | 137 (67.2) | *χ²*(2) = 2.28 | *p* = .320 |
| Education, years, *M* (%) | 13.5 (2.30) | 13.9 (1.80) | 14.2 (2.0) | *F*(2,320) = 2.47 | *p* = .086 |
| Social support, *n* (%) | 51 (86.4) | 48 (80.0) | 170 (83.3) | *χ²*(2) = 0.89 | *p* = .662 |
| BPRS^a^, *M* (SD) | 22.2 (4.20) | 23.0 (3.10) | - | *t*(117 )= −1.20 | *p* = .231 |
| Illness duration^a^, *M* (SD) | 13.5 (12.50) | 11.6 (8.70) | - | *t*(117) = 1.00 | *p* = .319 |
| Antidepressant use^a^, *M* (SD) | 25 (43.10) | 27 (28.30) | - | *χ²*(1) = 3.19 | *p* = .094 |

**Additional file 1. Supplementary analyses for variables with non-significant group differences**

Note.

Values are presented as mean (SD) or n (%).

p-values indicate overall group effects derived from one-way ANOVA for continuous variables and *χ²* tests for categorical variables.

Patient-only variables were compared between M and ST using independent-samples t tests or *χ²* tests, as appropriate.

M group, mood disorders group; ST group, subthreshold group; CC, community controls; BPRS, Brief Psychiatric Rating Scale.

^a^Patients only.
